# Supplementary material for: Content-rich biological network constructed by mining PubMed abstracts
Source: BMC Bioinformatics. 2004 Oct 8;5:147. doi: 10.1186/1471-2105-5-147 (PMC528731; doi:10.1186/1471-2105-5-147)
Supplement: Additional File 2 — The original results of the above study (non-essential files are deleted to keep the file size under the limit set by BMC bioinformatics). [file 1471-2105-5-147-S2.bz2 › chilibotAdditionalFile2/dip50/404ID8955093E10064/html/LIFR_OSM.html]

 


 **LIFR** and **OSM** 
  
Found 43 abstracts in PubMed, retrieved 43.  
 

 What does Google say? 
 PDF only 
| .edu only 

---

**Interactive relationship** (e.g. stimulation, inhibition, etc)

**Inhibitory relationship**- The cytokine receptor subunits gp130, leukemia inhibitory factor receptor  [ **LIFR** ]  alpha LIFRalpha, and oncostatin M  [ **OSM** ]  receptor beta OSMRbeta transduce  **OSM**  signals that regulate gene expression and cell proliferation.  Ref: 11602599 J Biol Chem, 2001
- Oncostatin M  [ **OSM** ]  binds the high affinity leukemia inhibitory factor receptor  [ **LIFR** ] .  Ref: 1536831 New Biol, 1992
- Leukemia inhibitory factor LIF, cardiotrophin 1, ciliary neurotrophic factor, and oncostatin M  [ **OSM** ]   **OSM**  lead to heterodimerization of LIF receptor  **LIFR**  or the  **OSM**  specific receptor OSMR with glycoprotein gp 130, the common receptor subunit for IL 6 type cytokines.  Ref: 10586060 J Immunol, 1999
- Leukemia inhibitory factor had no effect on hASMCs, indicating that  **OSM**  effects on hASMCs were mediated by the  **OSM**  type II receptor and not by the leukemia inhibitory factor receptor  [ **LIFR** ] .  Ref: 10590238 Circ Res< MedlineTA>Circ Res, 1999
- A biologically active  **OSM**  receptor has been previously described that consists of a heterodimer of leukemia inhibitory factor receptor  [ **LIFR** ]   **LIFR**  and gp130.  Ref: 8999038 J Biol Chem, 1996
- Oncostatin M  [ **OSM** ]  regulates the synthesis and turnover of gp130, leukemia inhibitory factor receptor  [ **LIFR** ]  alpha, and oncostatin M  [ **OSM** ]  receptor beta by distinct mechanisms.  Ref: 11602599 J Biol Chem, 2001
- Using neutralizing antibodies to gp 130, the  **OSM**  receptor OSMR, and the leukemia inhibitory factor receptor  [ **LIFR** ]   **LIFR** , we document that  **OSM**  exclusively uses the OSMR gp 130 heterodimer in signaling events, rather than the  **LIFR**  gp 130 heterodimer.  Ref: 10899931 J Neurochem, 2000
- The leukemia inhibitory factor LIF, cardiotrophin 1 CT 1 and oncostatin M  [ **OSM** ]   **OSM**  are four helix bundle cytokines acting through a common heterodimeric receptor composed of gp130 and LIF receptor  **LIFR** .  Ref: 12707269 J Biol Chem< MedlineTA>J Biol Chem, 2000
- The low affinity leukaemia inhibitory factor receptor LIF R  [ **LIFR** ]  is a component of cell surface receptor complexes for the multifunctional cytokines leukaemia inhibitory factor, ciliary neurotrophic factor, oncostatin M  [ **OSM** ]  and cardiotrophin 1.  Ref: 9396734 Biochem J, 1997
- The leukemia inhibitory factor receptor  [ **LIFR** ]  LIF R  [ **LIFR** ]  subunit is a component of cell surface receptor complexes for the multifunctional cytokines, LIF, cardiotrophin 1, ciliary neurotrophic factor, and human oncostatin M  [ **OSM** ] .  Ref: 11042511 Leuk Lymphoma, 2000
- Ciliary neurotrophic factor CNTF, leukemia inhibitory factor LIF, oncostatin M  [ **OSM** ]   **OSM** , and interleukin 6 IL6 compose a family of distantly related cytokines that initiate signaling by inducing either homodimerization of the beta signal transducing receptor component gp130 in the case of IL6 or heterodimerization between gp130 and the gp130 related  **LIFR**  beta in the case of CNTF, LIF, and  **OSM** .  Ref: 7512571 J Biol Chem, 1994
**Neutral relationship**- Both mouse and human  **OSM**  engaged gp130 with their respective OSMRbeta subunits, but only human  **OSM**  also acted through  **LIFR** .  Ref: 10854424 J Biol Chem, 2000
- The amount of  **LIFR**  was down regulated in both LIF and  **OSM**  producing cells and this effect was not found in wild type U373 MG cells treated with externally added cytokines.  Ref: 11284716 Biochem J, 2001
- Our results demonstrate that both LIF and  **OSM**  cause tyrosine phosphorylation and activation of the gp130. **LIFR**  combination, but the gp130.OSMRbeta complex is activated by  **OSM**  only.  Ref: 8999038 J Biol Chem, 1996
- **OSM**  appears, therefore, to act as a weak agonist of LIF dependent processes on murine cells with a 10 fold lower specific activity than that of LIF, which is in agreement with human  **OSM**  cross reacting with the murine LIF R  [ **LIFR** ] .  Ref: 8050491 Exp Cell Res, 1994
- Our results indicate that LIF, CT 1 and  **OSM**  share an overlapping binding site located in the Ig like domain of  **LIFR** .  Ref: 12707269 J Biol Chem< MedlineTA>J Biol Chem, 1994
- The different behavior of LIF and CT 1, on one side, and of  **OSM** , on the other side, can be related to the different affinity of their site III for  **LIFR** .  Ref: 12707269 J Biol Chem< MedlineTA>J Biol Chem, 1994
- The functional receptor for LIF is composed of  **LIFR**  alpha chain LIFRalpha and gp130 both of which are shared in the functional receptors for oncostatin M  [ **OSM** ] , ciliary neurotrophic factor, and cardiotrophin 1.  Ref: 9852103 J Biol Chem, 1998
- Oncostatin M  [ **OSM** ]  can activate two related receptors, one consisting of a low affinity LIF receptor subunit,  **LIFR**  beta, which forms a heterocomplex with the gp130 signal transducing protein and can recognize both oncostatin M  [ **OSM** ]  and LIF, and a second heterocomplex consisting of a subunit that specifically recognizes oncostatin M  [ **OSM** ] , OSMR beta, and the gp130 protein.  Ref: 12061840 Cytokine, 2002
- Recombinant human Oncostatin M  [ **OSM** ]  rhOSM can induce the transient tyrosine 705 phosphorylation of STAT3 in serum starved  **LIFR**  beta OSMR beta expressing ovarian carcinoma cell lines, but does not alter cell growth and effects only a modest increase in the apoptotic rate in these cultured cells.  Ref: 12061840 Cytokine, 2002
- An antagonist for the leukemia inhibitory factor receptor  [ **LIFR** ]  inhibits leukemia inhibitory factor, cardiotrophin 1, ciliary neurotrophic factor, and oncostatin M  [ **OSM** ] .  Ref: 9341130 J Biol Chem, 1997

**Non-interactive relationship** (e.g. studied together, co-existance, homology, etc.)

- To define the specificity of the signal transduction by the LIF  **OSM**  receptor a heterodimer of gp130 and LIF receptor  **LIFR**  and the  **OSM**  specific receptor a heterodimer of gp130 and  **OSM**  receptor OSMR, we reconstituted the receptor function by transfection into receptor negative Hep3B hepatoma cells.  Ref: 9182534 J Biol Chem, 1997
- gp130 either homodimerizes in response to IL 6 and IL 11 or forms heterodimers with the leukemia inhibitory factor LIF receptor  **LIFR**  in response to LIF, oncostatin M  [ **OSM** ]   **OSM** , ciliary neurotrophic factor CNTF, cardiotrophin 1 CT 1 or cardiotrophin like cytokine resulting in the onset of cytoplasmic tyrosine phosphorylation cascades.  Ref: 12047380 Eur J Biochem, 2002
- Continuous treatment with Dex 10 8 mol L inhibited the endogenous production of IL 6, LIF,  **OSM** , IL 11R, and OSMR, but had no detectable effect on the expression of IL 11, CT 1, CNTF, CNTFR,  **LIFR** , or gp130.  Ref: 12110437 Bone, 2002
- Flow cytometric analyses detected both the  **OSM**  specific receptor and the gp130 receptor at the chondrocyte cell surface, but failed to detect the leukemia inhibitory factor receptor  [ **LIFR** ]   **LIFR** .  Ref: 11465713 Arthritis Rheum, 2001
- The Y759 motif in gp130 also attenuates gene induction mediated by the oncostatin M  [ **OSM** ]  and leukemia inhibitory factor receptor  [ **LIFR** ]  complexes, which both contain gp130 as the shared subunit.  Ref: 10946280 J Immunol, 2000
- All of these cell lines express the receptor components of  **OSM**  and leukemia inhibitory factor LIF gp130,  **LIFR**  and the  **OSM**  specific OSMRbeta.  Ref: 11038252 Brain Res Mol Brain Res, 2000
- gp130, the leukemia inhibitory factor receptor  [ **LIFR** ]  LIFRbeta and the  **OSM**  specific receptor OSMRbeta.  Ref: 11811789 Growth Factors, 2001
- Eighteen pituitary adenoma biopsies removed in transsphenoidal surgery six corticotrophic adenomas, four nonfunctioning adenomas, four somatotrophinomas, four prolactinomas and six normal anterior pituitaries were examined for the expression of IL 6 receptor R, leukemia inhibitory factor LIF, LIF R  [ **LIFR** ] , IL 11, IL 11 R, oncostatin M  [ **OSM** ]   **OSM** ,  **OSM**  R, ciliary neurotrophic factor CNTF, CNTF R and cardiotrophin 1 CT 1.  Ref: 11095488 J Clin Endocrinol Metab, 2000
- On cells that also express  **OSM**  receptor,  **OSM**  was not antagonized, demonstrating that the antagonist is specific for LIF R  [ **LIFR** ] .  Ref: 9341130 J Biol Chem, 1997
- METHODS Active transcription of LIF, IL 6, and  **OSM** , their related receptors LIF R  [ **LIFR** ] , IL 6 R, and gp130, and the consecutive signal transducing molecules STAT 1, STAT 3, and STAT 5a were analyzed in reverse transcriptase polymerase chain reaction experiments.  Ref: 9488310 J Neurosurg, 1998
- The expression of all other cytokines LIF, IL 11, CNTF and receptors IL 6 R, LIF R  [ **LIFR** ] , IL 11 R,  **OSM**  R, CNTF R was found in different patterns in the adenoma subtypes and normal pituitaries.  Ref: 11095488 J Clin Endocrinol Metab, 2000
- Its mechanism of action is via specific binding to gp130 and either the leukaemia inhibitory factor receptor  **LIFR**  or oncostatin M  [ **OSM** ]  receptor OSMR systems at the cell surface to form an active signalling complex.  Ref: 10997905 Structure Fold Des, 2000
- A549 epithelial cells are subjected to growth inhibition only in the presence of  **OSM**  ED50 = 6 ng ml, even though they expressed LIF R  [ **LIFR** ]  and gp130 transcripts.  Ref: 8050491 Exp Cell Res, 1994
- Using RT PCR we detected transcripts of the receptor components involved in  **OSM**  signaling, gp130,  **OSM**  receptor OSMR beta, and leukemia inhibitory factor receptor  [ **LIFR** ]   **LIFR** , in HCEC.  Ref: 11706938 J Neuropathol Exp Neurol, 2001
- Contributions of leukemia inhibitory factor receptor  [ **LIFR** ]  and oncostatin M  [ **OSM** ]  receptor to signal transduction in heterodimeric complexes with glycoprotein 130.  Ref: 10586060 J Immunol, 1999
- We observed the expression of mRNA transcripts for  **OSM** ,  **OSM**  receptor subunit beta, leukaemia inhibitory factor receptor subunit  **LIFR** , and glycoprotein 130 in endometrial and endometriotic stromal cells.  Ref: 11420390 Mol Hum Reprod, 2001
- Since LIF together with LIFRalpha appear to form a more stable complex with gp130 than  **OSM**  with gp130 and OSMRbeta, co activation of  **LIFR**  and OSMR resulted in a predominant LIF like response.  Ref: 10854424 J Biol Chem, 2000
- The leukemia inhibitory factor receptor  [ **LIFR** ]  LIF R  [ **LIFR** ]  is activated not only by LIF, but also by cardiotrophin 1, ciliary neurotrophic factor with its receptor, and oncostatin M  [ **OSM** ]   **OSM** .  Ref: 9341130 J Biol Chem, 1997
